# Pathway to Equity in Water, Sanitation, and Hygiene (WASH) in Africa: Challenges and opportunities

**DOI:** 10.1097/EE9.0000000000000315

**Published:** 2024-06-21

**Authors:** Adetoun Mustapha, Temitope Laniyan, Andrew Reigns, Wondwossen Birke, Firdu Zawide

**Affiliations:** a Nigerian Institute of Medical Research, Yaba, Lagos, Nigeria; b Department of Environmental Health Sciences, Faculty of Public Health, College of Medicine, University of Ibadan, Ibadan, Oyo State Nigeria; c Federal Polytechnic Ugep, Cross River State, Nigeria; d Jimma University Department of Environmental Health Science & Technology, Jimma, Ethiopia

**What this study adds**

This commentary provides an assessment of the progress made on the implementation of Water, Sanitation, and Hygiene services in Africa. It highlights the constraints and opportunities and proffers insights on how to build resilient pathways to increase the speed of implementation and achieve universal coverage of Water, Sanitation, and Hygiene services in Africa based on not just study findings but also the lived experiences of the symposium participants. It brings the topic to the front burner of the global health community to stimulate advocacy and actions that will ensure that vulnerable populations in Africa are not left behind in achieving Sustainable Development Goal 6.

## Introduction

Water, sanitation, and hygiene are essential to health and life. Therefore, access to safe water, basic sanitation, and good hygiene practices not only keeps the population healthy but also helps the society to thrive by increasing education, productivity, job creation, and economic development.^1^

On 28 July 2010, the United Nation General Assembly adopted resolution A/RES/64/292, which recognizes the human right to safe and clean drinking water and sanitation.^2^

According to the 2022 WHO/UNICEF Joint Programme Monitoring Report on Water, Sanitation, and Hygiene (WASH) in Africa, it is estimated that around 411 million people lack basic water services, 779 million people lack access to basic sanitation, and 839 million lack basic hygiene. Two out of three people in urban areas lack safely managed sanitation, including 193 million who practice open defecation. The report also revealed that about 30% of the schools sampled had no access to basic water and sanitation services. Only about 51% of health facilities have basic water services, and 22% have access to basic sanitation.^3^ The United Nations Sustainable Development targets 6.1 and 6.2 refer to universal and equitable access to drinking WASH for all by 2030.^4^ This implies all settings, including households, schools, health care facilities, workplaces, and public spaces.^4,5^ Thus, access to basic WASH services remains very low in Africa. By global standards, there are wide disparities between and within countries, between the rural poor and the urban rich.^6,7^

The millions of Africans who drink polluted water and live in unhealthy environment are exposed to the risk of a number of preventable diseases such as diarrhea, cholera, dysentery, typhoid, and parasitic diseases that cause illnesses, death, and stunted lives.^8^ Diarrhoeal disease is the second leading cause of death in children under 5 years old.^9^ Access to WASH can also impact schooling and time spent collecting water from distant sources. Several studies from the region confirm that women and girls suffer disproportionately from the lack of adequate WASH services. They bear the burden of water collection over long distances, which has been associated with negative effects on well-being, school attendance, and a higher risk of gender-biased violence.^10^

During the International Society for Environmental Epidemiology (ISEE) 2021 Annual Conference, the Africa Chapter sponsored a symposium to discuss progress made to eliminate the inequalities and constraints in WASH in Africa, with talks from 4 African regions. It describes the benefits of investing in WASH, the regional disparities for WASH service, the current level of investment, and the roles of national governments, their international development partners, and the ultimate users, the general public including the rural and urban poor. It further identifies changes in the approach of delivering WASH service by shifting from the business-as-usual mentality to practical action based on need assessment. The abstracts of the talks are available at: https://www.iseepi.org/docs/ISEE-2021-Abstract-E-Book.pdf.

## Insights from the symposium

The key insights on challenges and the opportunities toward achieving equity in WASH in Africa from the presentations were: (1) strengthening collaboration among stakeholders and more investment in WASH programs; (2) building on past achievements and involving local communities in design and implementation of WASH programs; (3) capacity building, institutional strengthening, and local production of WASH equipment; (4) review sector policy and transfer WASH data and information into knowledge asset; (5) strengthen institutional collaboration and coordination; and (6) develop realistic financing strategies to achieve target.

## Strengthening stakeholders collaboration and more investment in WASH

Stakeholders at the forefront of the WASH program in Africa include the United Nations’ system and member states, multilateral agencies, nongovernmental organizations (NGOs), civil societies, the private sector, governments, and local communities. Significant efforts by the stakeholders have built partnerships since the first Water and Sanitation Decade (1981–1990) to make access to clean water and sanitation available to all people in the world.^11^ Most countries have been successful in raising awareness both in governments and among external support agencies, NGOs, and the general public to solicit support for their effort to expand WASH services to the poor rural villages, periurban areas, and urban areas that are exposed to health risks.^12^ Although the coverage of WASH in Africa has shown steady improvement over the years, the situation is less favorable with regard to the rural and urban poor creating a great inequality both in water and sanitation services.^6,7,13^ Reports from the countries showed that they are now facing constraints that hinder progress. These constraints are natural, demographic, cultural, social, political, technical, institutional, and financial.^14^ Africa has more than enough water resources to meet the needs of its people but the resource is not evenly distributed across the continent.^15^ Climate change is also a threat to water resource development in Africa as it is reducing rainfall and increasing drought and water scarcity.^16^ More than 25% of the world’s refugees live in Africa exerting additional pressure for the provision of WASH services in refugee camps.^17^ Urbanization in Africa is also expected to reach 60% by 2050 due to massive internal displacement of people from rural to urban areas attributed to extreme climate conditions, political and ethnic conflicts, and economic opportunity.^18^ Evidence shows that increasing urbanization in Africa is not always accompanied by similar growth in job opportunities, and this has increased the economic burden to make WASH available to the poor unemployed city dwellers and migrants who live in financial hardship.^6,19^

There has been a significant reduction in external funding over the years, and efforts by some African countries to meet their water and sanitation needs have been less than what is expected under Sustainable Development Goal 6 (SDG 6).^1,3,4,19^ Data on investments into the water and sanitation sector in Africa showed that more investment was made both from national and external sources for the urban sub-sector, leaving behind those who are in greater need of the service: the rural, periurban, and urban poor.^20,21^ Accelerating WASH coverage in Africa will require countries to be in the driver’s seat of the WASH program and strengthening collaboration and coordination of stakeholders at all levels (local, national, regional, and international). It will also require greater government allocation to WASH as evidence of political commitment, more funding and knowledge transfer from external support agencies and international NGOs, more private sector investment in WASH, and promoting culturally acceptable WASH infrastructure and development of applicable financing mechanisms for its maintenance and sustainability by the local communities.^1,4,14,20^

## Building on past achievement and involving local communities in design and implementation of WASH program

Since the declaration of the International Drinking Water and Sanitation Decade (1981–1990) African countries have made steady progress in narrowing the gap in equity by directing solutions to their critical WASH problems.^11^ Although inequalities persist, the international commitment remains a hope for the poor and a backbone for national governments. Many lessons have been learnt from past efforts in the areas of resource mobilization, community participation, application of best practices, including the use of appropriate technology, monitoring and evaluation, operation and maintenance, and community management to mention a few.^22^ Yet instead of building on the success by improving and expanding what has been already developed and tested on the ground as a pilot project, new approaches or technologies are introduced without evidence for its suitability and acceptability.

Many pilot projects that have been successfully implemented in the past have not been scaled and some have been abandoned due to lack of maintenance and absence of community participation at the inception of the project. The WHO initiative on Participatory Hygiene and Sanitation Transformation (PHAST) program could be cited as an effective innovative approach to enable communities to identify their WASH needs, draw up plans in partnership to improve their environmental health condition by raising awareness, solicit support from local and external sources, and participate in the operation and maintenance of the WASH infrastructure.^23^ Countries in East and Southern Africa carried out pilot projects. PHAST helped communities improve their hygiene practices and manage their own water and sanitation program.^24,25^ In addition, extensive documentation of the methods and progress of the innovation was disseminated so that its success could be replicated elsewhere in the region. However, PHAST has not been widely replicated across Africa.

Some participants at the ISEE 2021 symposium shared their lived experiences on community participation in the WASH program. These include a lack of consultation of community members when water and sanitation facilities were built and “donated’’ to their communities. Hence, in some cases, facilities that are not compatible with local cultural practices were provided. This creates lack of ownership of these facilities by the communities leading to lack of maintenance and abandonment in the long run. In countries where participatory approach is adopted, communities have taken full management of their WASH program by training local artisans, bill collectors, and service attendants. These activities are guided and monitored by community water and development committees. This makes the WASH program more productive, acceptable, and realistic. It has also helped in the sustainability of the program, especially by ensuring self-reliance. Therefore, the achievement of the SDG 6 requires the involvement of local communities. Local communities should be included from the onset of the program, from planning to implementation, operation, maintenance, monitoring, and evaluation.

## Capacity building, institutional strengthening, and local production of WASH equipments

Accelerating WASH coverage in Africa requires the construction of a large number of WASH infrastructure and facilities that are accessible and affordable to the poor in rural, periurban, and urban areas. The lack of skilled personnel and professionals in the WASH sector has been reported by many countries as one of the constraints in implementing their national action plans.^26^ Human resources in terms of both quality and quantity are a concern for advancing the progress of WASH in Africa.^26^ Training for community management of rural WASH services has been going on for some time in some countries. The training covers the essentials in rural WASH management, construction of basic WASH infrastructure, operation and maintenance of basic WASH facilities, monitoring, general health, and hygiene education.^26^ The training of high-level professionals specialized in environmental and civil engineering, water resource management, financial analysts, urban water and sewerage system operators, and laboratory technicians is limited due to the lack of adequate institutions and high cost.^26,27^ Some countries still depend on external consultants and contractors to design and construct large urban water and sanitation projects.^26^ As a result, the cost of urban WASH service continues to escalate. Therefore, there is a need for cost-efficient measures. One way of achieving this is by strengthening the existing training and research institutions to train more professionals and engage in research activities that will improve the delivery of WASH services by developing appropriate technologies, improving the monitoring system and disease surveillance, water quality control, developing investment plan, and financing strategies. The allocation of funds for training from every large WASH project budget remains a step in the right direction and should be actively pursued by the countries.

Further, the lived experience shared by some participants at the ISEE 2021 symposium is that imported WASH equipments and chemicals are raising the cost of maintenance and replacement as the systems dilapidates over time. There are challenges of scarcity of spare parts due to the rapidly changing technology. Hence the need for local innovation to address the overdependence on importation of WASH equipment. With the rising cost of urban utilities, more effort is needed by the countries to reduce costs by developing local capacity to manufacture WASH equipment and to use local raw materials for these.

## Review sector policy and transfer data and information into knowledge asset

WASH policy should be pro-poor, demand-driven, inclusive of gender equity, and addressing the needs of the vulnerable population. The policy should be based on the information and analysis gathered from past assessment reports to help sharpen the focus on removing critical constraints and formulate regulations and legislation to move SDG 6 forward.^21^ National health policies in sub-Saharan African countries highlight that disease prevention is the guiding principle for improving public health rather than treating illnesses to cure the sick.^14^ However, the policies are often not translated into concrete actions when allocating the budget to environmental health and medical services. As Dr. Haldan Mahler, former director general of the World Health Organization (WHO), has said, “The number of water taps per 1000 persons is a better indicator of health than the number of hospital beds”.^28^ WASH is not only beneficial to health but contributes to livelihoods, school attendance, and dignity and helps to create resilient communities living in healthy environments.^3,9^ Investment in WASH must be considered as a cost-effective strategy to build a resilient health system.

There is the absence of an effective data monitoring system for parameters such as surveillance of waterborne diseases, water quality standard, and health and economic impact assessment due to investing on WASH at local and national level.^29^ The availability of more accurate data that includes health and economic outcomes will help decision-makers to prioritize financing for the WASH sector.^29,30^ There are several guidelines, training modules, and field reports on projects related to community management, gender inequalities, appropriate technology, micro-financing, operation and maintenance, and participatory hygiene and sanitation transformation in the United Nations system.^3,12,14,24^ The harmonization and dissemination of these valuable information and documents to training institutions, and sectoral ministries will be helpful in the transfer of knowledge and building expertise of practitioners, decision-makers, and the local communities and develop a knowledge asset.

## Strengthen institutional collaboration and coordination

Generally, WASH program comprises a very fragmented sector in Africa in terms of institutional responsibilities with many ministries and parastatal organizations in charge of water or sanitation services at different levels. Many ministries, public organizations, NGOs, international organizations, civil societies, and the private sector that are partners in WASH services are sometimes working in silos.^20,31^

Some countries have been successful in establishing interministerial and interagency national action committees to coordinate the activities of the different partners in implementing national action plans for universal access to WASH in their respective countries. Similar strategies have been followed for rural areas by mobilizing the communities and establishing village WASH committees which helped in the transfer of the rural WASH projects to community-based management. An example is the “ONE WASH National Program” in Ethiopia.^32^ The program is a joint initiative of the Ministry of Health, Water, Education and Finance promoted by UNICEF and several other partners engaged in WASH activities at all levels. Countries that have adopted the ONE WASH approach are making significant progress in addressing the needs of the rural poor by promoting community management, including cost sharing, hygiene education, use of appropriate technology, and local materials. On the contrary, very little progress is made when institutions are working in silos.^32,33^ Government agencies and parastatals in charge of WASH should carry out joint planning, financing, implementation, monitoring, and evaluation under ONE WASH project or program with the assistance of external partners. A number of countries have been successful in implementing their national action plan as a result of establishing effective coordination mechanisms and adopting the ONE WASH approach to make WASH available to the population at all levels.^32–34^

## Develop realistic financing strategies to achieve target

From the earliest years of the international drinking water and sanitation decade declaration, it was realized that the success in achieving targets depended largely on the commitment of all stakeholders.^11^ The United Nations, as an advocate for the cause, facilitated the buildup of partnership with external support agencies within and outside the UN system to support countries in achieving their targets. Evidence shows that countries are making significant efforts to achieve their objective of ensuring the availability of WASH services to those in greater need, the rural and urban poor in the framework of SDG 6.^3^ According to a report from African Ministers of Finance WASH service delivery has constrained by a financing gap at all levels, including international assistance and commitments which declined sharply from US$ 3.8 billion to US $ 1.7 billion in 2017 in Sub-Saharan Africa. Total current investments must be tripled to an annual amount of $ 11.4 billion, requiring six times the current rate of national government spending on the WASH sector.^35^ The African Finance Ministers have also agreed to collaborate with sector ministries to ensure that WASH investment plans and financing mechanisms are realistic and well-designed to achieve national targets. There are three sources of funding for WASH identified by the Finance ministers, user tariffs and fees allocation of taxes and revenues, and aid funding transfer. The need for transparency of WASH fund management and improved monitoring and evaluation of WASH programs have been noted.^35^

## Recommendations

Our symposium recommends the following to accelerate the WASH services coverage in Africa:

1. Review existing sector policy and strategy including the standards and the legislation for enforcement of the policy. The policy should focus on new approach to break away from business as usual. The UN SDG 6 directives are a useful reference.
2. Strengthen the database for WASH monitoring and documentation of best practices on community management, operation and maintenance of rural water supply, sanitation and hygiene facilities, micro-financing, cost recovery, training modules, and other useful tools to transform data to knowledge.
There is a need for strong advocacy to convince decision-makers that investment on WASH is an investment on health and economic development. This is based on cost-benefit analysis studies which showed that every $1 invested on WASH provides up to $7 economic return from lower health costs, more productivity, and fewer premature death.^36^ Ministries of health in particular need to review their budget allocation to preventive health care including environmental health.
3. Investment plans and financing strategies for the national WASH program should be realistic and well-designed to achieve targets in the national action plan in every country of the region. This requires high political commitment, increasing partnership of national and external support agencies, establishing effective collaboration and coordination mechanisms, capacity building, strong community participation, local manufacturing of wash equipment, institutional strengthening, innovative financing, transparency of fund management and accountability.

## Conclusions

In conclusion, achieving SDG 6 in Africa is a huge challenge due to a number of constraints that are natural, cultural, social, demographic, technical, financial, and so on. Every country should be committed to significantly increasing its service coverage through national leadership and global partnerships. Thus, establishing effective organizations and strengthening existing institutions within countries to implement sustainable WASH services that are culturally acceptable, manpower training, research, and local innovation are needed to build resilient pathways that will ensure that “no one is left behind” by 2030.
